# Selective serotonin reuptake inhibitors and inflammatory bowel disease; Beneficial or malpractice

**DOI:** 10.3389/fimmu.2022.980189

**Published:** 2022-10-06

**Authors:** Mohammad Reza Hatamnejad, Shaghayegh Baradaran Ghavami, Marzieh Shirvani, Mona Asghari Ahmadabad, Shabnam Shahrokh, Maryam Farmani, Ghazal Sherkat, Hamid Asadzadeh Aghdaei, Mohammad Reza Zali

**Affiliations:** ^1^ Basic and Molecular Epidemiology of Gastrointestinal Disorders Research Center, Research Institute for Gastroenterology and Liver Diseases, Shahid Beheshti University of Medical Sciences, Tehran, Iran; ^2^ Faculty of Pharmacy, Shiraz University of Medical Sciences, Shiraz, Iran; ^3^ Faculty of Medicine, Guilan University of Medical Sciences, Guilan, Iran; ^4^ Medicine Faculty of Mashhad Branch, Islamic Azad University, Mashhad, Iran; ^5^ Gastroenterology and Liver Diseases Research Center, Research Institute for Gastroenterology and Liver Diseases, Shahid Beheshti University of Medical Sciences, Tehran, Iran

**Keywords:** inflammatory bowel diseases, serotonin uptake inhibitors, pro-inflammatory, anti-inflammatory, Crohn’s disease, ulcerative colitis, antidepressant, dysbiosis

## Abstract

IBD, a chronic inflammatory disease, has been manifested as a growing health problem. No Crohn’s and Colitis councils have officially ratified anti-depressants as a routine regimen for IBD patients. However, some physicians empirically prescribe them to rectify functional bowel consequences such as pain and alleviate psychiatric comorbidities. On the other side, SSRIs’ prescription is accompanied by adverse effects such as sleep disturbances. Prolonged intermittent hypoxia throughout sleep disturbance such as sleep apnea provokes periodic reductions in the partial oxygen pressure gradient in the gut lumen. It promotes gut microbiota to dysbiosis, which induces intestinal inflammation. This phenomenon and evidence representing the higher amount of serotonin associated with Crohn’s disease challenged our previous knowledge. Can SSRIs worsen the IBD course? Evidence answered the question with the claim on anti-inflammatory properties (central and peripheral) of SSRIs and illuminated the other substantial elements (compared to serotonin elevation) responsible for IBD pathogenesis. However, later clinical evidence was not all in favor of the benefits of SSRIs. Hence, in this review, the molecular mechanisms and clinical evidence are scrutinized and integrated to clarify the interfering molecular mechanism justifying both supporting and disproving clinical evidence. Biphasic dose-dependent serotonin behavior accompanying SSRI shifting function when used up for the long-term can be assumed as the parameters leading to IBD patients’ adverse outcomes. Despite more research being needed to elucidate the effect of SSRI consumption in IBD patients, periodic prescriptions of SSRIs at monthly intervals can be recommended.

## Highlights

- Tissue-available dosage can be assumed as Serotonin’s affinity determinant in binding with receptors to represent the stimulatory or inhibitory functions.
- Complications associated with long-term SSRI use, such as dysbiosis, overwhelm its anti-inflammatory properties and stimulate the gut toward inflammation.
- Periodic prescription at monthly intervals is recommended to inhibit the biphasic dose-dependent serotonin behavior and adverse effects of long-term SSRI consumption.

## 1 Introduction

One of the most prevalent manifestations of chronic inflammation is Inflammatory Bowel Disease (IBD), which principally comprises Crohn’s Disease (CD) and Ulcerative Colitis (UC). IBD affects the colon, small intestine, or both and is characterized by chronic recurrent bowel ulceration (1). The IBD pathogenesis likely involves the complex interaction between genetic, environmental, and immunological factors resulting in an upsetting and exaggerated intestinal inflammatory response to intestinal microbiota in vulnerable individuals (2, 3). A significant part of disability and malfunctioning that occurs in chronic health problems is associated with psychological disorders (4).

Psychological disorders can affect the symptomatic disease courses and increase inflammation. Anxiety and depression are known as comorbidities in IBD patients (5). They are related to the Hypothalamic-Pituitary-Adrenal (HPA) axis activation and increased circulating cortisol levels. Lengthened activation of the HPA axis, as occurs in prolonged stress or chronic inflammation, including IBD, causes chronic cortisol level elevation, leading to reduced sensitivity of glucocorticoid receptors. Reduced glucocorticoid receptor sensitivity can enhance immunological responses and augment inflammation (6, 7). On the other hand, remedies with corticosteroids can induce psychiatric symptoms (7).

The findings declare that the depression prevalence is between 15% and 25%, with possibly lower rates in UC patients than in those who suffer from CD (8, 9). Anxiety is even more rampant, with rates of nearly 30% in IBD patients (10). The rates of anxiety and depression have been higher pending periods of disease flare-up (11). Interestingly almost three-fourths of anti-depressant medications are prescribed without companioning psychiatric indications (12). Approximately 30% of IBD sufferers administrate anti-depressant medications for mental health, bowel symptoms, or both (13).

Despite the NICE guideline, which suggests taking anti-depressants for the long term when depression is accompanied by a chronic physical ailment such as IBD or cancer (14), none of the Crohn’s and Colitis councils have officially ratified anti-depressants as a routine regimen for IBD patients. However, some physicians empirically prescribe them for two aims (15). First, they recommend anti-depressants to rectify functional bowel consequences such as pain (16). A qualitative study appraising the use of anti-depressants for IBD patients demonstrated that most gastroenterology specialists (78%) had treated the patients for symptoms palliation with anti-depressants as adjunctive therapy, especially for pain (17). Second, they claim anti-depressants profitability since they can ameliorate the psychiatric comorbidities in IBD (18).

However, findings regarding the direct benefit of anti-depressants on IBD, regardless of their impact on psychiatric comorbidities, are restricted, and existing ones are controversial, especially with current investigations. Hence, in this review, the molecular mechanisms and clinical evidence are scrutinized and integrated to clarify the proper decision-making about Selective Serotonin Reuptake Inhibitor (SSRI) prescribing. This study focused on SSRIs among all the anti-depressant classes since each class’s proficiency differs. SSRIs have been the most common anti-depressants since 1974 (19) when they were introduced, so debating on this group is more necessary. Functions and pathways should be discussed to recognize better what is happening during the SSRI treatment in IBD.

## 2 SSRI and IBD

### 2.1 Serotonin metabolism

**Figure 1:**
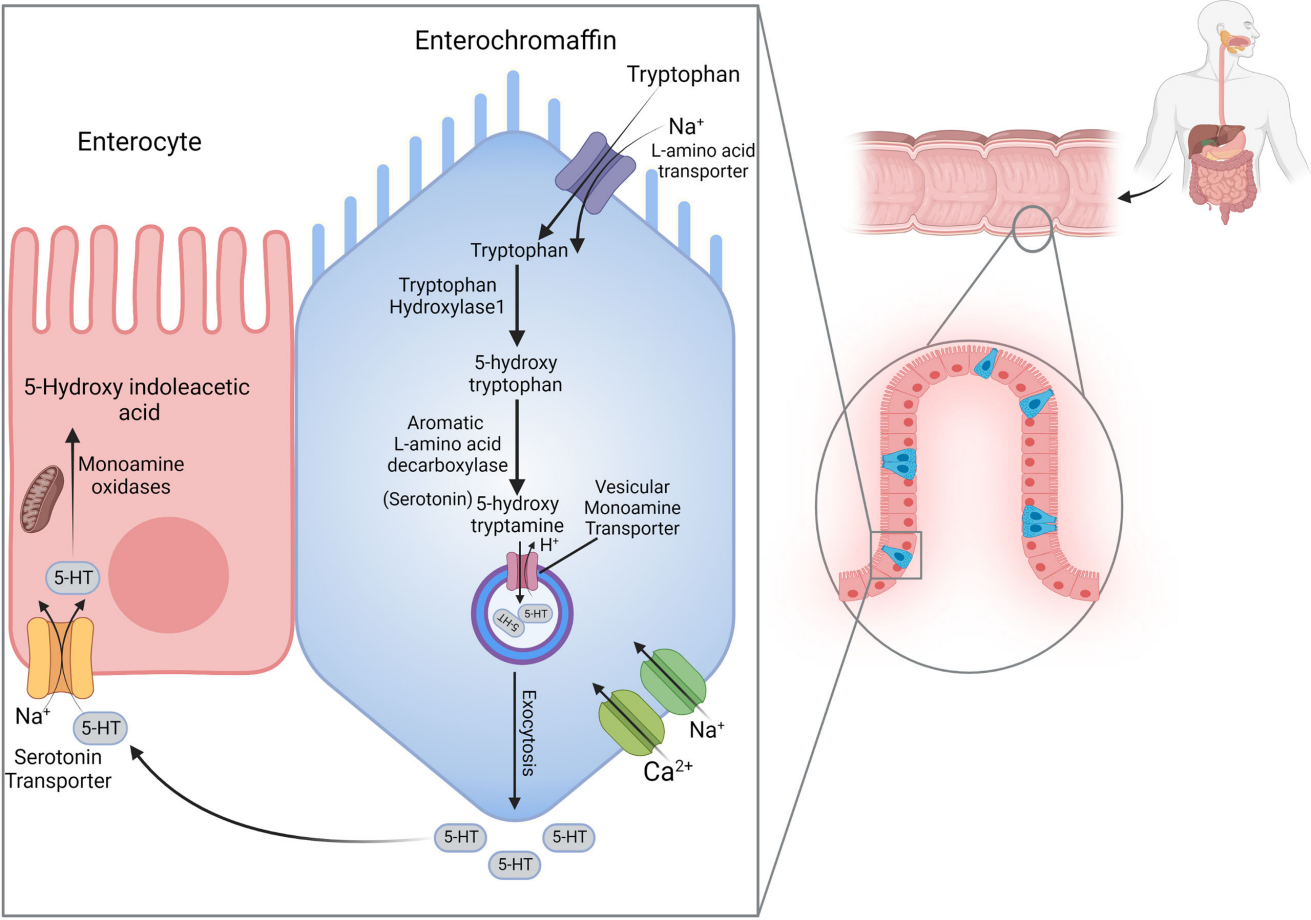
Serotonin Metabolism. Tryptophan intracellular transport is carried out by an L-amino acid transporter; then, it turns to 5-hydroxytryptophan, which is facilitated by tryptophan hydroxylase-1. In the second step, aromatic L-amino acid decarboxylase converts 5-hydroxytryptophan into 5-hydroxytryptamine; eventually, serotonin is stored in vesicles by Vesicular Monoamine Transporter. With the influx of Ca^2+^, serotonin is exocytosed into the extracellular matrix. After affecting the targeted receptors, it is reabsorbed by the serotonin transporter and degraded by Monoamine oxidases to 5-hydroxyindoleacetic acid.

The gut and brain, constructing 95% and 5% of serotonin (5-Hydroxytryptamine or 5-HT), respectively, are considered the primary sources of serotonin in the body. Serotonergic neurons generate serotonin in the central nervous system (CNS); however, this process in the gut is conducted by enterochromaffin (EC) cells and the myenteric nerve plexus (20). Considering that an enormous quantity of serotonin is attributed to the EC cells, 5-HT metabolism and homeostasis are narrated with a focus on EC cells (**Figure 1**).

#### 2.1.1Production

Serotonin is synthesized from the amino acid L-tryptophan (Trp) within two stages. At first, with the indole ring hydroxylation, tryptophan is converted to 5-hydroxytryptophan (5-HTP) (21). This action is mainly catalyzed by tryptophan hydroxylase (TPH), a rate-limiting enzyme identified as two isoforms (22). TPH1 originates in the pineal gland and gut, and TPH2 is located in the brain and serotonergic enteric nerves (23, 24). Within the next stage, amino acid decarboxylation is taken place by tryptophan decarboxylase (25). The final product, 5-HT, is stowed inside the vesicles by the vesicle-associated transporter (21).

#### 2.1.2 Distribution and function

By the luminal stimuli, mechano- and chemo-sensitive ion channels on the EC cells are activated, and, with the influx of Ca^2+^ ions, 5-HT is discharged from the basolateral and apical surfaces of EC cells (26, 27). Intraluminal-released 5-HT can affect gut microbiota’s composition and function (28), whereas serotonin in tissue-surrounding basal space acts as a pleiotropic component. 5-HT exerts diverse operations in the gastrointestinal (GI) tract through distinct receptors. Serotonin receptors can be divided into seven types (15 subtypes) from 5-HT1 to 5-HT7, which are all families of G-protein-coupled receptors. In contrast, 5-HT3 is a member of the nicotinic/Gamma-aminobutyric acid (GABA_A)_ family of Na^+^/K^+^ channel proteins (29, 30). Except for 5-HT5 and 5-HT6 receptors, other classes can be found in the GI tissue (31). Mucosal secretion is adjusted with the effect of serotonin on epithelial 5-HTR2 and neuronal 5-HTR1P, 5-HTR3, and 5-HTR4 receptors (32). Besides, the impact of 5-HT3R and 5-HT4R agonists to reinforce muscular peristalsis has illuminated the motor regulatory role of 5-HT (26). Platelets’ serotonin transporter (SERT) can pick up released extracellular serotonin. 5-HT is a component of the clotting process (31); thus, platelets are the blood serotonin reservoirs that liberate the 5-HT to develop vasoconstriction during bleeding (33).

#### 2.1.3 Expunction

After all effects on the enterocytes, myenteric neurons, and leukocytes in the surrounding tissue and catching up by platelets remain accessible 5-HT should be deactivated. Serotonin is re-uptaken by the SERT, and monoamine oxidase incepts a process in which 5-HT eventually turns into 5-hydroxy indoleacetic acid (34). SERT exists on both enterocytes and presynaptic neurons; For this reason, to terminate the serotonin function, expunction can be performed within either EC cells-surrounding basal space or synaptic cleft (31, 35). Here is where exactly SSRIs act and, with the serotonin re-uptaking blockage, 5-HT consequences are continued (36).

### 2.2 Adverse effects of SSRIs on IBD

#### 2.2.1 GI Upset

GI upset can be a common concern of individuals with IBD and is reported with many anti-depressant medications. These side effects are dose-dependent and tend to decrease over the first weeks of treatment. As a symptom of IBD, diarrhea was reported more often with sertraline than with the rest of the SSRIs medicines (7).

#### 2.2.2 Risk of the bleeding

Mesalazine [5-aminosalicylic acid (5-ASA)] has been used for a long time to treat IBD. It is an effective, safe, well-tolerated remedy for mild to moderate UC (37, 38). SSRI use per self has been associated with an increased risk of bleeding, particularly during the administration’s first month. The inhibition of SERT by SSRIs is supposed to be responsible for the risk of bleeding. Platelets release serotonin at the bleeding site. They do not synthesize 5-HT but acquire it from the blood and store it (39). In this way, SSRIs may also worsen the bleeding caused by ASA, the backbone of Mesalazine. The inhibition of cytochrome P450 by SSRIs has also been associated with an increased risk of drug interaction, exacerbating the bleeding (40). This risk may be increased by continuous synchronous use of other medications associated with an increased risk of bleeding (41).

#### 2.2.3 Sleep disturbances

Different adverse effects have been identified with SSRIs, including sleep disturbance, sexual dysfunction, and weight gain. The most troubling ones are seen during long-term SSRI medication (42). Sleep Apnea (SA), as a sleep disturbance category, is linked with many comorbidities, leading to generally augmented morbidity. The negative effect of sleep-disordered breathing and its connection with some comorbidities are supposed to be secondary to the recurrent hypoxia and sleep destruction that characterize this disorder (43).

##### 2.2.3.1 SSRIs, sleep disturbances, and dysbiosis

A concept that seems to be interconnected to numerous ailments is the gut microbiome. Human microbiota refers to the broad and thorough gathering of microbes living in an organ such as the gut. Changes in gut microbiota have been presented as critical in developing some chronic diseases (44). Simultaneously, changes in the gut microbiome are related to SA, implying that the gut microbiome is an ordinary player in SA. Prolonged intermittent hypoxia (IH) throughout sleep provokes periodic reductions in the partial oxygen pressure (PO2) gradient in the gut lumen, promoting changes in the relative plenty of aerobic bacteria alongside the rise of amplified obligate and facultative anaerobes (45).

Moreover, such conversion can be achieved through the direct impact of SSRIs on the gut luminal bacterium (46, 47). SSRIs can exert antimicrobial activity and affect bacterium community diversity, especially considering they are taken for long durations (48, 49). One theory proposed for SSRIs antimicrobial relics is that they may undergo passive diffusion across the phospholipid membrane more efficiently, allowing interaction with cellular machinery (50). Also, SSRIs have been shown to inhibit microbial efflux pumps, which contribute to antimicrobial resistance and include competitive and non-competitive inhibition and clogging of the pump’s external pore (51). In addition, Diviccaro et al. have recently displayed that commencing or withdrawing paroxetine (a class of SSRI) treatment can directly alter the gut microbiota composition (52). However, for SSRIs to affect microbiota diversity, they would need to be present in the gut lumen at sufficient concentrations, especially in the microbial-enriched regions such as the ascending colon (53).

##### 2.2.3.2 How does dysbiosis augment the inflammation and lead to IBD?

Pieces of evidence struggle to identify the possible mechanisms through which gut dysbiosis contributes to many comorbid medical circumstances such as IBD. Some bacterial species, within the normal gut microbiota composition, display mucin-degrading features and, at the same time, do the fermentation of nutritional fibers. This leads to short-chain fatty acids (SCFA) production, which are vital nutrients and energy sources for the colon epithelium (54). Gut dysbiosis reduces butyrate and acetate levels, depriving the epithelium of needed nutrients, which can cause epithelium dysfunction (55–57). In addition, IH, which represents recurrent reoxygenation cycles, induces epithelium damage (58). These perturbations cause disorders in the tight junctions among the intestinal epithelial cells (59). As one of the manifestations of alteration in gut microbiota baseline composition, toxin-producing bacteria may be increased. Endotoxins produced by these germs can efficiently translocate through the damaged gut epithelium into the systemic circulation and cause a state of systemic inflammation (**Figure 3**) (60). Moreover, a subsidiary duty of butyrate as the regulator of T-cells differentiation is affected by nutrition poverty, promoting inflammation (61).

With dysbiosis, the expression of genes contributing to oxidative stress mechanisms will be enhanced (62). Sulfate-reducing bacteria level elevation in UC results in hydrogen sulfate production, which can cause intestinal mucosal inflammation due to cytotoxic features (63). Other dysbiosis-mediated IBD elements are linked with Trp metabolism (**Figure 3**). Trp metabolism with three distinct pathways touches the immune system. Indole derivatives manufactured by the aryl hydrocarbon (Ahr) signaling pathway modulate immune balance (e.g., regulation of killer T-cells) (64). Kynurenine (Kyn) secondary production due to the Kyn pathway exhibits inflammation regulation responses (65). Serotonin, the final component of 5-HT signaling, has a crucial role in immune reactions (66), which will be explained in the subsequent section.

Abnormal regulation of 5-HT in the human gut has been implicated with various GI disorders, such as IBD (67, 68). Pieces of evidence showed that 5-HT was increased in CD (69, 70) and serum serotonin enhanced with SSRIs; therefore, our former perception of SSRIs’ advantages comes across some questions. Can SSRIs worsen the IBD course by inducing SA or providing a high amount of 5-HT? Despite these detrimental effects of SSRIs in IBD patients, for which purposes are they prescribed for these individuals?

### 2.3 Useful effects of SSRIs on IBD

Investigators formerly believed that serotonin-dependent mechanisms constitute a small part of IBD pathogenesis, but other substantial elements are responsible for it. Thus, an increase in 5-HT content due to SSRIs therapy cannot be mainly related to IBD. On the other hand, relieving IBD symptoms boosts the patients’ tolerance (16, 71), eliminating psychiatric comorbidities and reducing relapse rates (11, 72). The direct anti-inflammatory trace of SSRIs (73, 74) encouraged physicians to prescribe SSRIs.

#### 2.3.1 SSRI and anti-inflammation

The discovery of the inflammation role in the pathogenesis of depression proposed that anti-depressants may participate in inflammatory mechanisms and immune system regulation (75). A meaningful correlation between psychological stress and increased inflammation affirmed the mood disorders-inflammation association (76). A significant decrease in serum C-reactive protein concentrations four weeks after initiating SSRIs in people with major depressive disorder (77) corroborated their immune-regulatory properties. SSRIs do not significantly impact inflammatory mechanisms, but other factors modulate the complex interaction between SSRIs and inflammation (78). Although both anti-inflammatory (73, 74, 79, 80) and pro-inflammatory (75, 81–84) effects of SSRIs were reported, prescribing them seems to be based on their anti-inflammatory nature.

**Figure 2:**
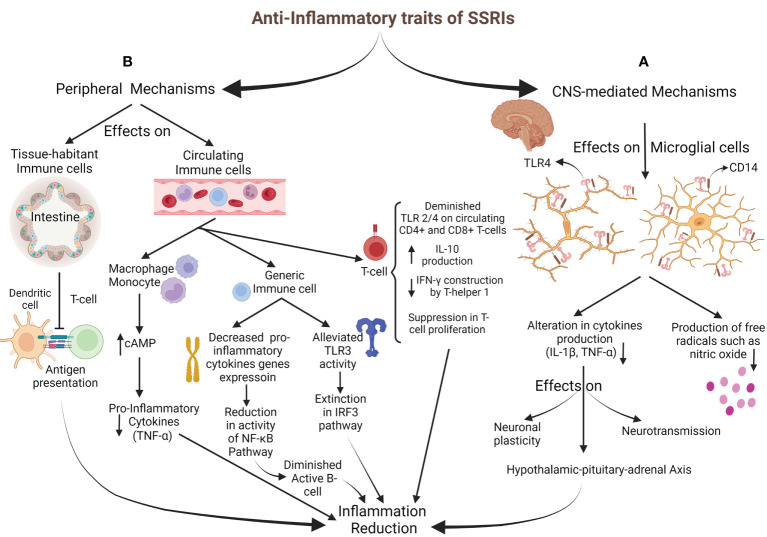
Anti-Inflammatory properties of SSRIs. **(A)** CNS-mediated mechanisms: SSRIs target microglial cells to impact through TLR4 and CD14, resulting in decreased inflammatory cytokines generation (IL-1ß and TNF-α) and reduced free radicals production. HPA-axis activation *via* alteration in cytokine network comes up with inflammation decrement. **(B)** Peripheral mechanisms: T-cell activation is inhibited by diminished antigen presentation of intestine-habitant DC. Circulating immune cells are also impressed; increasing cAMP in monocytes and macrophages brings about inflammatory cytokines drop. Generic immune cells were targeted through decreased pro-inflammatory cytokine gene expression, followed by subsidence in the NF-κB pathway activity and B-cell activation. Also, alleviating TLR3 activity with the inhibition of the IRF3 pathway leads to an inflammation drop. SSRIs directly affect T-cell by reducing TLR2/4 expression, a decline in IFN-γ generation, a rise in IL-10 production, and disturbance in their proliferation.

The anti-inflammatory effects of SSRIs can be categorized into two classes based on the mechanism’s mediators: CNS-mediated mechanisms, represented by microglial cells, and peripheral mechanisms, facilitated by tissue-habitant or circulating immune-system cells (**Figure 2**).

##### 2.3.1.1 CNS-mediated mechanisms

Microglial cells are the targets for SSRIs and Serotonin and norepinephrine reuptake inhibitors in CNS-mediated mechanisms to apply their anti-inflammatory impresses (84). They are essential in inflammatory regulation and primary inflammatory response (85, 86). The glial cells mainly express Toll-Like Receptor (TLR) 4 and CD14. TLRs mediate immune responses to exogenous and endogenous stimulations and are also vital regulators for neuroimmune reactions caused by stress and major depression. CD14 plays a co-receptor role for TLR4 when inflammatory responses release inflammatory factors like tumor necrosis factor-α (TNF-α) and interleukin (IL)-1ß (87–90). Different cytokines (such as TNF-α and IL-1ß) are released by activated glial cells to influence neurotransmission, HPA activity, neuronal plasticity, and neurogenesis. Previous studies have suggested that anti-depressant drugs act through altered cytokine networks (91). SSRIs have been shown to influence microglia’s capability to produce pro-inflammatory cytokines and free radicals, including nitric oxide (82, 92–94). In their conventional pharmacological dosage, Tynan et al. reported that SSRIs (fluoxetine, sertraline, paroxetine, fluvoxamine, and citalopram) with the impression of TLR decreased TNF-α production by microglia. However, they have established that SSRIs were moderate pro-inflammatory agents when used for lengthened periods at low doses (95).

##### 2.3.1.2 Peripheral mechanisms

The peripheral mechanisms mainly influence the immune system’s regulation and inflammatory cytokine production. A recent study in multiple sclerosis patients with major depression reported that the expression of TLR2 and TLR4 on circulating CD4^+^ and CD8^+^ T-cells decreased with SSRI therapy, reducing cytokine production (96). In addition, medications such as sertraline may debar the inflammation *via* the TLR3-IRF3 pathway (97). So, SSRI therapy in the stressful condition is associated with anti-inflammatory responses (98–100). Another suggested SSRI anti-inflammatory process is mediated by their possible effects on decreasing nuclear factor kappa-light-chain-enhancer of activated B cell (NF-κB) activity (101), which is induced by the expression of pro-inflammatory cytokine genes (14, 102). The other suggested mechanism is through the cyclic adenosine monophosphate (cAMP). Vetulani et al. declared that anti-depressants could increase intracellular cAMP (103) in microglia and macrophages, alleviating pro-inflammatory cytokines (104, 105). Another anti-inflammatory mechanism is the inhibition of interferon-gamma (IFN-γ) production by T helper 1. Also, SSRIs suppress mitogenic-stimulated T-cell proliferation and increase IL-10 (a cytokine against inflammation) production by T-cells (81). Diminished expression of inducible co-stimulatory ligand on intestinal dendritic cells (DCs) by fluoxetine down-modulates the antigens presenting from DCs to T-cells and impedes constitutes of inflammatory responses against gut microbiota (106). Stated specificities against the inflammatory processes are serotonin-independent, and the performance of SSRIs *via* 5-HT will be described in another section. Besides the anti-inflammatory properties of SSRIs, other components can justify their prescription.

## 3 Clinical evidence

The advantage of fundamental science is more when it becomes clear that the outcomes can be generalized to clinical science. Assumed mechanisms about the usefulness of SSRIs should experiment with under clinical conditions (**Table 1**).

**Table 1:** Clinical documents of SSRI prescription.

Authors (year)	Study design	Sample population	Result
**Supporting evidences**			
Kristensen et al. (2019) (107)	Prospective cohort	42,890 cases	Anti-depressants in UC and CD patients lead to decreasing the relapse rate and the risk of taking corticosteroids and anti-TNF.
Macer et al. (2017) (108)	Systematic review	N/A	The vast majority of studies (80%) concluded the effectiveness of anti-depressant therapy in IBD patients.
Yanartas et al. (2016) (109)	Prospective cohort	67 cases	Six-month anti-depressant therapy improved depression, anxiety, and sexual function, as well as the disease activity.
Dehghanzadeh et al. (2015) (110)	Randomized controlled trial	44 cases	Effectiveness and safety of anti-depressants for treatment of depression and anxiety and decreased Lichtiger Colitis Activity Index.
Iskandar et al. (2014) (111)	Retrospective observational study	81 cases	The effectiveness of anti-depressants in gastrointestinal symptom relief based on the Likert response score.
Goodhand et al. (2012) (72)	Retrospective observational study	29 cases	The advantages of antidepressants in reduction of relapse rate, corticosteroid dependency, and endoscopy procedures.
Mikocka-Walus et al. (2009) (112)	Systematic review	N/A	Antidepressants posse a positive influence on the course of IBD.
**Dissenting evidences**			
Blackwell et al. (2021) (113)	Prospective cohort	6373 cases	Continued SSRI use is associated with steroid dependency and demonstrated no beneficial effect on the UC course.
Frolkis et al. (2019) (114)	Retrospective cohort	403,665 cases	No discrepancy in the prevention of IBD in the trial of SSRI vs. placebo within the healthy population.
Mikocka-Walus and Andrews (2014) (115)	Cross-sectional	98 cases	Most IBD patients reported no improvement in their symptoms following the SSRI treatment.
Bonderup et al. (2014) (116)	Case-control	5751 cases	The positive association between SSRIs and microscopic colitis.
Fernández-Bañares et al. (2007) (117)	Case-control	233 cases	SSRIs increase the hazard of microscopic colitis
**Impartial evidence**			
Cochrane Database (2019) (16)	Systematic Review And Meta-analysis	N/A	No firm decision regarding the efficacy and safety of antidepressants in IBD patients can be made

### 3.1 Supporting evidence of SSRI prescription

Goodhand et al. described that prescribing anti-depressants (including SSRIs) to remedy concomitant depression in IBD patients not only ameliorates the current course of inflammation but also, by preventing inflammation relapse, diminishes the need for corticosteroid therapy and endoscopy (72). Daghaghzade et al., in a randomized, double-blind controlled clinical trial, demonstrated the significant profit of duloxetine in improving the frequency of diarrhea and severity of symptoms like pain in IBD patients (110). Also, the amending effects of SSRIs on inflammation and symptoms of IBD patients were affirmed by the CD activity index, as illustrated by Yanartas et al. (109). Another prospective cohort study conducted between 2000 and 2017 with around 44,000 subjects indicated a positive aspect of anti-depressant treatment in IBD patients, particularly those with no history of taking anti-depressants before IBD diagnosis. A better influence was found in CD patients compared to UC patients. Thus, this study showed that patients treated with anti-depressants had a significantly lower risk of receiving corticosteroids and anti-TNF medication than patients who did not take anti-depressants (107). In agreement with previous findings, Iskandar et al., in a retrospective cohort investigation, represented that the anti-depressants can appease the severity of IBD patients’ condition, more specifically in UC patients (111) (**Table 1**).

### 3.2 Evidence against SSRI prescriptions

In a qualitative questionnaire-based online survey by Mikocka-Walus et al., most IBD patients observed no difference with taking the SSRIs, and only 25% declared an enhancement in their clinical presentations (115). Exploring the relationship between SSRIs and inflammation in the colon led to detecting a significant positive association. Fernandez et al. realized that patients with microscopic colitis (both types of lymphocytic and collagenous) possessed a higher rate of taking SSRIs than the control group, particularly in the case of sertraline (117). In a sizeable case-control study with 5,751 microscopic colitis cases based on nationwide Danish registries, Bonderup et al. figured out a positive association between SSRI exposure and microscopic colitis (116). In a retrospective cohort study conducted from 1986 to 2012 on 400,000 patients with new-onset depression, SSRIs were indicated as protective agents for UC and CD development in depressed patients. However, uncertainty was raised since they had found no discrepancy in IBD development in the trial of SSRI vs. placebo among normal individuals. SSRIs cannot provide their protective role when consumed due to any indication except depression (prevention of IBD in the healthy population) (114).

Despite documents about SSRIs’ advantages in IBD patients being controversial, comprehensive systematic reviews announced that SSRIs are beneficial for IBD courses as the conclusion (108, 112). Certainty about prescribing SSRIs for IBD management existed until 2021 when Blackwell et al. declared that continued administration of SSRIs or tricyclic antidepressants (TCA) is a red flag for IBD patients. They indicated that continuous consumption of SSRIs or TCAs is correlated with corticosteroid dependency and worse clinical outcomes in the future (113). The power of their study, comprising a large sample size (over 6,000 participants with UC) and long-term follow-up (between 2005 and 2016), challenged our previous belief about SSRIs. Also, this prospective cohort study reinforces the conclusion of a Cochrane systematic review in 2019 that declared no firm decision regarding the profits of SSRIs (16). How could these consequences be justified with all our knowledge about SSRIs? Basic science answers.

## 4 Behind the scene

**Figure 3:**
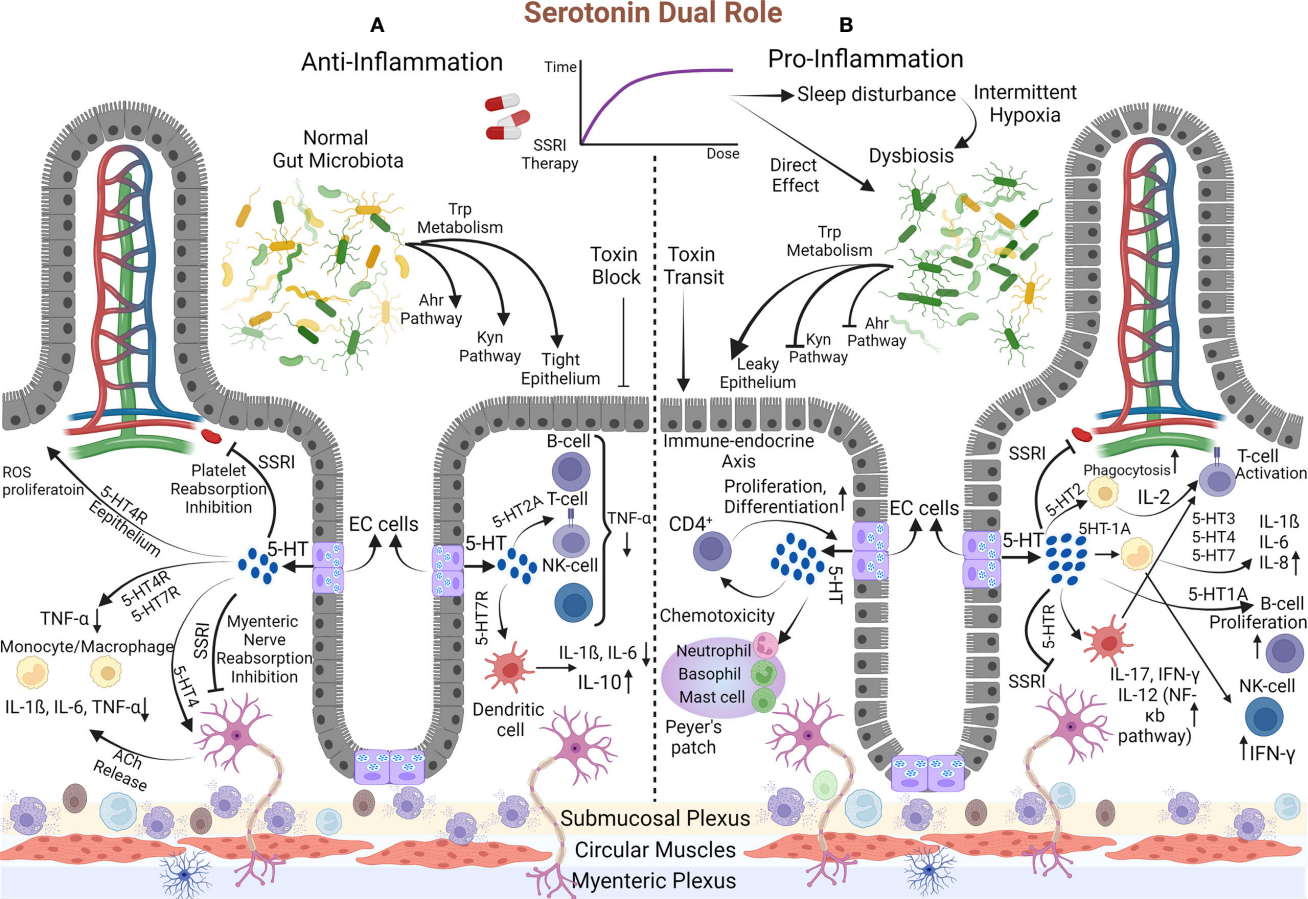
The dual demeanor of Serotonin. **(A)** Anti-Inflammatory: The gut microbiota enter the Trp into Ahr and Kyn pathways and the 5-HT synthesis process. Initially, SSRIs inhibit 5-HT reabsorption in platelets and myenteric nerves and boost mucosal serotonin content, initiating anti-inflammatory mechanisms. Proliferation and resistance to ROS arise in the epithelium *via* the 5-HT4 receptor. 5-HT reduces TNF-α production by monocytes and macrophages (1) directly through their 5-HT4 and 5-HT7 receptors and (2) indirectly *via* the 5-HT4 receptor on the myenteric nerve, which is accompanied by releasing Ach that eventually comes up with IL-1ß, IL-6, and TNF-α decrement. Similar changes in TNF-α fabrication occur *via* B-cell, T-cell, and NK cell 5-HT2 receptors. Additionally, 5-HT7 on the DC turns down the IL-6 and IL-1ß and elevates IL-10. **(B)** Pro-inflammatory: Over time, SSRI dosage augmentation affects, directly and indirectly, the gut microbiota composition, and disrupts Ahr and Kyn pathways. Pathogens’ toxins pass the leaky epithelium created by dysbiosis and stimulate the gut toward inflammation. Moreover, the inhibition of 5-HT reabsorption is amplified, resulting in much more serotonin levels. This primes pro-inflammatory mechanisms, which are known as the endocrine-immune axis. Macrophage phagocytosis is reinforced, and through the 5-HT2 receptor, they release IL-2 that activates T-cells. In addition, by acting on DC, 5-HT activates T-cells and increases IL-17, IL-12, and IFN-γ *via* the NF-κB pathway. Monocyte 5-HT1A receptor provocation reduces the inhibitory effect on NK-Cell to increase IFN-γ production. On the other hand, the impacts on 5-HT3, 5-HT4, and 5-HT7 monocyte receptors increase IL-6, IL-8, and INF-1ß. Also, B-Cell proliferates and activates through 5-HT1A receptor instigation. The serotonin amount can boost neutrophil, basophil, and mast cell chemo-toxicity. This process is turned into a destructive cycle since increased T-cells (CD4^+^ types) initiate the immune-endocrine axis (EC cells proliferation and boosting the serotonin content). Eventually, the destructive cycle exacerbates the inflammation.

Previous evidence made the point clear that desired outcomes of prescribing SSRIs are free from their effects on 5-HT and rely on their immune-regulatory properties; nevertheless, subsequent clinical proof made us doubtful about the utility of these medications. Why should these adverse consequences appear in IBD patients after years of SSRI consumption? We hypothesized that an abundant amount of serotonin resulting from continuous consumption of SSRIs with the effect on immune system induction might overwhelm the anti-inflammatory SSRIs’ features. We need to scrutinize the exact interactions between 5-HT and the immune system for this theory (**Figure 3**).

### 4.1 Striking role of serotonin in the immune system

Recognizing serotonin as a pivotal component in the immune system began with discovering serotonergic receptors on immune cells in 1982 (118, 119). Since then, some immune system functions have been attributed to serotonin, recognized as the endocrine-immune axis. Serotonin can enhance macrophage phagocytosis (120), increase DC, basophil, neutrophil, and mast cell chemotaxis (121), and regulate cytokine production (122, 123). Lately, it was elucidated that monocytes, macrophages, and T-cells not only can be assumed as peripheral sources of 5-HT (124, 125) but also can induce the immune-endocrine axis by affecting EC cells. Likewise, Wang et al., in a model to discover the manner of the immune-endocrine axis, compared the amount of 5-HT production between wild-type mice and severe combined immunocompromised mice in the setting of contamination with the same Trichuris muris infection. They revealed new interactions between CD4^+^ T cells with EC cells to enhance the generation of 5-HT in the gut *via* Th2-based mechanisms (126). Also, detecting the IL-13 receptor on EC cells reinforced previous findings (126).

### 4.2 Stimulatory or Inhibitory modulator

Despite the cyclic interaction between the immune-endocrine and endocrine-immune axis, serotonin can eventually be defined as an immune system stimulatory or inhibitory modulator (**Figure 3**).

As a stimulatory constituent, 5-HT actuates the molecular mechanisms toward inflammation. The proliferation of T-cells and B-cells is mediated by 5-HT2 and 5-HT1A receptors, respectively (127, 128), whereas T-cells are activated *via* their 5-HT7 receptors (129). Treated mice by P-chlorophenyl alanine (inhibitory molecule of TPH-1) indicated that with the effect of serotonin on macrophage 5-HT2 receptors, an accessory pathway to activate CD4^+^ cells by releasing IL-2 is initiated (130). Serotonin binding to the 5-HT1A receptor on monocytes makes these cells less efficient in suppressing NK cells that are typically inhibited. Thus, cytotoxicity and IFN-γ production will be augmented based on NK cell activity (131, 132). Later, evidence of enhancing the NK cell proliferation and their cytosolic functions was obtained by trials of SSRI medications (133). Another study declared that serotonin directly improves NK cell function while some dopamine/serotonin antagonists suppress the CD16-mediated activity of NK cells (134). Durk et al. demonstrated that the augmentations of IL-1ß, IL-6, IL-12p40, and IL-8/CXCL8 cytokines are 5-HT3, 5-HT4, and 5-HT7 monocyte receptor-mediated (135). Serotonin by activating DCs motivates CD4^+^ T-cells to generate IL-17 and IFN-γ cytokines (136). Studies on disease-specific cytokine patterns have elucidated the type of CD4^+^ T-cells differentiation and cytokine production. Th1 and Th17, with their manufactured cytokines (IFN-γ, IL-17, and IL-22), are assumed to be related to CD pathogenicity. Meanwhile, Th2-like differentiation with increased natural killer T-cells producing IL-13 is associated with UC (137).

The inhibitory role of releasing serotonin to decrease inflammation has been portrayed in other investigations. In an asthma model, the 5-HT2A receptor on eosinophil exerts its anti-inflammatory function by preventing the recruitment to the inflammatory site (138). Suppression of IFN-γ-inducing macrophage phagocytosis through 5-HT occurs at the high concentration of IFN-γ (at the physiological dose of IFN-γ, it provides stimulatory phagocytes) (139). Inhibition of TNF-α production by peripheral blood mononuclear cells was illustrated by 5-HT2A excitation (140). As another piece of evidence, TNF-α decrement was authenticated by 5-HT4 and 5-HT7 monocyte receptors instigation (135). In an *in-vitro* rat model, mosapride (5-HT4 agonist) administration enhanced acetylcholine (Ach) release from myenteric neurons, resulting in macrophages/monocytes Ach receptors activation. The expression of cytokines’ (IL-1ß, IL-6, and TNF-α) mRNA decreased in addition to immune cell recruitment suppression (141).

### 4.3 New insight into serotonin function

The dual role of serotonin not only can be exerted in the GI system but also can affect other organs such as the vasculature. Vascular smooth muscle cells synthesize IL-6, possibly inducing atherogenicity of vessels in response to serotonin (142). Furthermore, downregulating the expression of pro-inflammatory genes and preventing the TNF-α-mediated inflammatory pathways are obtained by selective activation of the 5-HT2A receptor on aortic smooth muscle cells (143).

The accumulative evidence pointed to the fact that performing the stimulatory or inhibitory role of 5-HT depends on the receptor and the cell to which it attaches. The 5-HT4 receptor on epithelial cells is responsible for the proliferation, resistance to Reactive Oxygen Species (ROS)-induced apoptosis, and eventually anti-inflammatory effect (based on barrier function improvement) (144). In contrast, in colitis mice, IL-6 and IL-1ß (responsible for inflammation) excessed with the administration of intraperitoneal 5-HT and its binding to the 5-HT4 receptor on immune cells (145). In addition to cell type, the receptor is another role-determining element. Activation of 5-HT7 receptors on antigen-presenting DCs and lipopolysaccharide-stimulated macrophages, likely with the cytokine production adjustment, diminishes inflammation severity (146). In contrast, by implicating the other receptor on the same cell (DC), serotonin leads to the elevation of IL-12 (through the NF-κB pathway), IL-17, IFN-γ, and ultimately the deterioration of inflammation (136).

No literature has discussed the serotonin’s affinity determinant factors in binding to receptors to represent the stimulatory or inhibitory function. It is now obscure that with the dissemination of 5-HT, why it operates excitatory and does not exert its prohibitory action, or conversely. A theory that may justify this phenomenon is dose-dependent serotonin behavior. This theory’s origin dates back to when Kubera et al. (95) discovered a dose-dependent binary 5-HT function in cytokine production. They believed cytokine production (IL-6 and TNF-α) by macrophages and lymphocytes needs low-level serotonin, whereas a high dose of serotonin reduces these cytokines; however, in our literature review, more convincing evidence illustrated a direct correlation between serotonin dose and stimulatory action. Tolerance induced by the SSRIs’ long-term use, giving rise to consumption dose increment. A high amount of available serotonin primes its stimulatory demeanor and steers the gut toward inflammation. This process, accompanied by the SSRIs’ dose-dependent functional alteration (shifting to pro-inflammatory performance when applied for long periods (74)), exacerbates the IBD patients’ condition.

The hypothesis of inhibitory or excitatory dose-dependent serotonin role was extended, one more time, by the serotonin-gut microbiota axis in which a lower amount of mucosal 5-HT with direct and indirect (via producing the antimicrobial peptides, specifically ß-defensins) effects on gut microbiota leads to reducing pro-inflammatory cytokines, enhancing the epithelial barrier function, and eventually prohibiting gut inflammation, and vice versa (147).

The theory of SSRI intake’s role in setting up an oxidative stress response has been debated (148, 149). Therefore, SSRI-induced oxidative stress can cause cellular damage and release endogenous ligands like adenosine triphosphate. These particles have been termed damage-associated molecular patterns (DAMPs), which are recognized by the pattern recognition receptors (PRRs) on immune cells like DC cells (150, 151). The role of PRR signaling in instigating the intestinal immune cells against the microbiota and inducing inflammation has been illuminated (152). Therefore, the act of SSRIs to cause the immune response, dysbiosis, and IBD through the PRRs can fortify the previous hypothesis.

## 5 Conclusion

Numerous studies have claimed SSRIs’ benefits due to their anti-inflammatory properties and psychiatric comorbidities treatment. In contrast, biphasic dose-dependent serotonin behavior accompanying SSRI shifting function, when used up for the long-term, can be assumed as the reason for IBD patients’ adverse outcomes. Despite more trials and cohorts being needed to illuminate the exact effect of long-term SSRIs consumption in IBD patients, periodic prescriptions of SSRIs at monthly intervals can be recommended.

## Author contributions

MZ, HA, and SS conceived the idea for the manuscript and, in cooperation with MH and SG, refined the latest theory. MH, MS, MA, and GS carried out the data mining and literature review. MH, MS, MA, and MF drafted the manuscript. MH, MS, and SG revised the manuscript. MH drew the figures. MZ, HA, and SS supervised the article preparation. All authors read and approved the final manuscript.

## Conflict of interest

The authors declare that the research was conducted in the absence of any commercial or financial relationships that could be construed as a potential conflict of interest.

## Publisher’s note

All claims expressed in this article are solely those of the authors and do not necessarily represent those of their affiliated organizations, or those of the publisher, the editors and the reviewers. Any product that may be evaluated in this article, or claim that may be made by its manufacturer, is not guaranteed or endorsed by the publisher.

## Abbreviations

IBD, Inflammatory bowel disease; CD, Crohn’s disease; UC, Ulcerative colitis; HPA, Hypothalamic-pituitary-adrenal; SSRI, Selective Serotonin Reuptake Inhibitors; 5-HT, 5-Hydroxytryptamine; CNS, central nervous system; EC, Enterochromaffin; Trp, Tryptophan; TPH, Tryptophan hydroxylase; GI: Gastrointestinal; GABA, Gamma-aminobutyric acid; SERT, Serotonin transporter; 5-ASA, 5-Aminosalicylic acid; SA, Sleep apnea; IH, Intermittent hypoxia; SCFA, Short-chain fatty acids; TLR, Toll-Like Receptor; TNF, Tumor Necrosis Factor; IL, Interleukin; NF-kB, Nuclear factor kappa-light-chain-enhancer of activated B cell; cAMP, cyclic Adenosine monophosphate; IFN-γ, Interferon-gamma; Ahr, Aryl hydrocarbon; Kyn, kynurenine; TCA, Tricyclic Antidepressant; DC, Dendritic Cell; Ach, acetylcholine; ROS, reactive oxygen species.
